# (*E*)-3-(4-Chloro­phen­yl)-1-(2,4-dichloro-5-fluoro­phen­yl)prop-2-en-1-one

**DOI:** 10.1107/S1600536808012178

**Published:** 2008-05-03

**Authors:** Hoong-Kun Fun, Suchada Chantrapromma, P. S. Patil, M. S. Karthikeyan, S. M. Dharmaprakash

**Affiliations:** a X-ray Crystallography Unit, School of Physics, Universiti Sains Malaysia, 11800 USM, Penang, Malaysia; b Department of Chemistry, Faculty of Science, Prince of Songkla University, Hat-Yai, Songkhla 90112, Thailand; c Department of Studies in Physics, Mangalore University, Mangalagangotri, Mangalore 574 199, India; d Syngene International Pvt Limited Plot No. 2 & 3 C, Unit-II Bommansandra Industrial Area, Bangalore 99, India

## Abstract

In the title chalcone derivative, C_15_H_8_Cl_3_FO, the dihedral angle between the two benzene rings is 43.35 (8)°. Weak C—H⋯O and C—H⋯Cl intra­molecular inter­actions involving the enone group generate *S*(5) and *S*(6) ring motifs, respectively. In the crystal structure, mol­ecules are linked into anti­parallel chains along the *a* axis. These chains are stacked along the *b* axis and short Cl⋯F contacts of 3.100 (1) Å link adjacent mol­ecules of the anti­parallel chains into dimers.

## Related literature

For hydrogen bond motifs, see: Bernstein *et al.* (1995 ▶). For bond-length data, see: Allen *et al.* (1987 ▶). For related structures, see, for example: Fun *et al.* (2007 ▶); Patil *et al.* (2007*a* ▶,*b*). For background to the applications of substituted chalcones, see, for example: Agrinskaya *et al.* (1999 ▶); Patil *et al.* (2006 ▶); Shivarama Holla *et al.* (2004 ▶). For related literature, see: Gu *et al.* (2008 ▶).
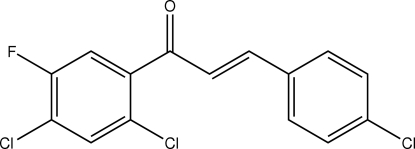


## Experimental

### 

#### Crystal data

C_15_H_8_Cl_3_FO
*M* *_r_* = 329.56
Monoclinic, 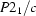

*a* = 6.8271 (1) Å
*b* = 3.7832 (1) Å
*c* = 52.0206 (10) Å
β = 96.100 (1)°
*V* = 1336.00 (5) Å^3^
*Z* = 4
Mo *K*α radiation
μ = 0.69 mm^−1^
*T* = 100.0 (1) K
0.35 × 0.29 × 0.18 mm

#### Data collection

Bruker SMART APEX2 CCD area-detector diffractometer
Absorption correction: multi-scan (*SADABS*; Bruker, 2005 ▶) *T*_min_ = 0.794, *T*_max_ = 0.889
42462 measured reflections
5852 independent reflections
5257 reflections with *I* > 2σ(*I*)
*R*_int_ = 0.036

#### Refinement

*R*[*F* ^2^ > 2σ(*F* ^2^)] = 0.051
*wR*(*F* ^2^) = 0.113
*S* = 1.28
5852 reflections
181 parameters
H-atom parameters constrained
Δρ_max_ = 0.47 e Å^−3^
Δρ_min_ = −0.31 e Å^−3^

### 

Data collection: *APEX2* (Bruker, 2005 ▶); cell refinement: *APEX2*; data reduction: *SAINT* (Bruker, 2005 ▶); program(s) used to solve structure: *SHELXTL* (Sheldrick, 2008 ▶); program(s) used to refine structure: *SHELXTL*; molecular graphics: *SHELXTL*; software used to prepare material for publication: *SHELXTL* and *PLATON* (Spek, 2003 ▶).

## Supplementary Material

Crystal structure: contains datablocks global, I. DOI: 10.1107/S1600536808012178/sj2488sup1.cif

Structure factors: contains datablocks I. DOI: 10.1107/S1600536808012178/sj2488Isup2.hkl

Additional supplementary materials:  crystallographic information; 3D view; checkCIF report

## Figures and Tables

**Table 1:** Hydrogen-bond geometry (Å, °)

*D*—H⋯*A*	*D*—H	H⋯*A*	*D*⋯*A*	*D*—H⋯*A*
C8—H8⋯Cl2	0.93	2.81	3.1164 (16)	101
C9—H9⋯O1	0.93	2.57	2.878 (2)	100

**Fig. 1.**
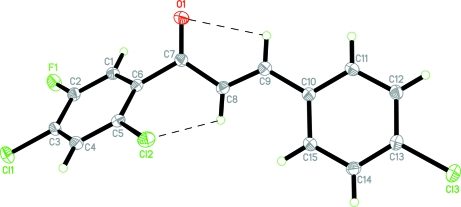
The asymmetric unit of (I), showing 50% probability displacement ellipsoids and the atomic numbering. Weak intramolecular C—H···O and C—H···Cl interactions are drawn as dashed lines.

**Fig. 2.**
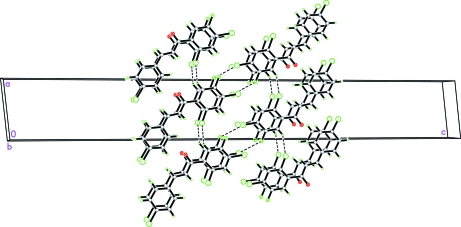
The crystal packing of (I), viewed along the b axis showing stacking of anti-parallel chains of molecules approximately along the b axis. Cl···F short contacts and weak C—H···O and C—H···Cl interactions are drawn as dashed lines.

## supplementary crystallographic information

### Comment

In recent years extensive research has been carried out on organic nonlinear optical materials particularly chalcone derivatives due to their high nonlinearity, varied synthesis, and better laser damage resistance as compared to their inorganic counterparts (Agrinskaya *et al.*, 1999; Shivarama Holla *et al.*, 2004; Patil *et al.*, 2006). In view of the importance of these organic materials, the title compound (I) was synthesized and its crystal structure is reported here.

The total molecular structure of the title compound (Fig. 1) is not planar, the dihedral angles between the two benzene rings is 43.35 (8)°. Atoms O1, C6, C7 and C8 lie on a plane and the least-squares plane through this moiety makes dihedral angles of 47.45 (10)° and 4.16 (10)° with the C1–C6 and C10–C15 benzene rings, repectively. The orientation of the prop-2-en-1-one unit can be indicated by the torsion angles C7–C8–C9–C10 = 177.37 (16)° and O1–C7–C8–C9 = 7.5 (3)°. Bond lengths and angles in (I) are in normal ranges (Allen *et al.*, 1987) and comparable to those in related structures (Fun *et al.*, 2007; Patil *et al.*, 2007*a*; 2007*b*).

In the structure, weak C9—H9···O1 and C8—H8···Cl2 intramolecular interactions generate S(5) and S(6) ring motifs (Bernstein *et al.*, 1995) (Table 1). In the crystal structure (Fig. 2), the molecules are linked into anti-parallel chains along the *a* axis. These chains are stacked along the *b*-axis and short Cl···F contacts of 3.100 (1) Å link adjacent molecules of the anti-parallel chains into dimers. The crystal is also stabilized by weak C—H···O and C—H···Cl intramolecular interactions (Table 1).

### Experimental

The title compound was synthesized by the condensation of 4-chlorobenzaldehyde (0.01 mol) with 2,4-dichloro-5-fluoroacetophenone (0.01 mol) in methanol (60 ml) in the presence of a catalytic amount of sodium hydroxide solution (10 ml, 10%). After stirring for 8 hr, the contents of the flask were poured into ice-cold water (500 ml) and left to stand for 5 hr. The resulting crude solid was filtered and dried. Colorless block-shaped single crystals of the title compound suitable for *x*-ray structure determination were recrystallized from acetone.

### Refinement

All H atoms were placed in calculated positions with d(C—H) = 0.93 Å, *U*_iso_=1.2*U*_eq_(C) for CH and aromatic atoms. The highest residual electron density peak is located at 0.67 Å from C4 and the deepest hole is located at 0.54 Å from Cl1.

### Crystal data

**Table:**

C_15_H_8_Cl_3_FO	*F*_000_ = 664
*M**_r_* = 329.56	*D*_x_ = 1.638 Mg m^−^^3^
Monoclinic, *P*2_1_/*c*	Mo *K*α radiation λ = 0.71073 Å
Hall symbol: -P 2ybc	Cell parameters from 5852 reflections
*a* = 6.8271 (1) Å	θ = 0.8–35.0º
*b* = 3.7832 (1) Å	µ = 0.69 mm^−^^1^
*c* = 52.0206 (10) Å	*T* = 100.0 (1) K
β = 96.100 (1)º	Block, colorless
*V* = 1336.00 (5) Å^3^	0.35 × 0.29 × 0.18 mm
*Z* = 4	

### Data collection

**Table:**

Bruker SMART APEX2 CCD area-detector diffractometer	5852 independent reflections
Radiation source: fine-focus sealed tube	5257 reflections with *I* > 2σ(*I*)
Monochromator: graphite	*R*_int_ = 0.036
Detector resolution: 8.33 pixels mm^-1^	θ_max_ = 35.0º
*T* = 100.0(1) K	θ_min_ = 0.8º
ω scans	*h* = −11→11
Absorption correction: multi-scan(SADABS; Bruker, 2005)	*k* = −6→6
*T*_min_ = 0.794, *T*_max_ = 0.889	*l* = −83→72
42462 measured reflections	

### Refinement

**Table:**

Refinement on *F*^2^	Secondary atom site location: difference Fourier map
Least-squares matrix: full	Hydrogen site location: inferred from neighbouring sites
*R*[*F*^2^ > 2σ(*F*^2^)] = 0.051	H-atom parameters constrained
*wR*(*F*^2^) = 0.113	*w* = 1/[σ^2^(*F*_o_^2^) + (0.0246*P*)^2^ + 1.777*P*] where *P* = (*F*_o_^2^ + 2*F*_c_^2^)/3
*S* = 1.29	(Δ/σ)_max_ = 0.001
5852 reflections	Δρ_max_ = 0.47 e Å^−^^3^
181 parameters	Δρ_min_ = −0.31 e Å^−^^3^
Primary atom site location: structure-invariant direct methods	Extinction correction: none

### Special details

**Table:**

Experimental. The low-temperature data was collected with the Oxford Cyrosystem Cobra low-temperature attachment.
Geometry. All e.s.d.'s (except the e.s.d. in the dihedral angle between two l.s. planes) are estimated using the full covariance matrix. The cell e.s.d.'s are taken into account individually in the estimation of e.s.d.'s in distances, angles and torsion angles; correlations between e.s.d.'s in cell parameters are only used when they are defined by crystal symmetry. An approximate (isotropic) treatment of cell e.s.d.'s is used for estimating e.s.d.'s involving l.s. planes.
Refinement. Refinement of *F*^2^ against ALL reflections. The weighted *R*-factor *wR* and goodness of fit *S* are based on *F*^2^, conventional *R*-factors *R* are based on *F*, with *F* set to zero for negative *F*^2^. The threshold expression of *F*^2^ > σ(*F*^2^) is used only for calculating *R*-factors(gt) *etc*. and is not relevant to the choice of reflections for refinement. *R*-factors based on *F*^2^ are statistically about twice as large as those based on *F*, and *R*- factors based on ALL data will be even larger.

### Fractional atomic coordinates and isotropic or equivalent isotropic displacement parameters (Å^2^)

**Table:**

	*x*	*y*	*z*	*U*_iso_*/*U*_eq_	
Cl1	0.75834 (7)	0.29526 (14)	0.494133 (8)	0.02272 (10)	
Cl2	0.27758 (6)	−0.21297 (12)	0.414214 (9)	0.01926 (9)	
Cl3	−0.33602 (6)	0.43796 (13)	0.273464 (9)	0.02136 (9)	
F1	1.03386 (16)	0.3790 (4)	0.45548 (2)	0.0240 (2)	
O1	0.75361 (19)	−0.1864 (4)	0.37069 (3)	0.0211 (3)	
C1	0.8267 (2)	0.1756 (5)	0.41951 (3)	0.0159 (3)	
H1	0.9260	0.2170	0.4090	0.019*	
C2	0.8591 (2)	0.2477 (5)	0.44562 (3)	0.0164 (3)	
C3	0.7139 (3)	0.1882 (5)	0.46193 (3)	0.0163 (3)	
C4	0.5343 (2)	0.0489 (5)	0.45181 (3)	0.0169 (3)	
H4	0.4363	0.0044	0.4625	0.020*	
C5	0.5022 (2)	−0.0237 (4)	0.42545 (3)	0.0147 (3)	
C6	0.6458 (2)	0.0408 (4)	0.40887 (3)	0.0144 (3)	
C7	0.6216 (2)	−0.0317 (5)	0.38025 (3)	0.0153 (3)	
C8	0.4414 (2)	0.0999 (5)	0.36537 (3)	0.0163 (3)	
H8	0.3552	0.2390	0.3737	0.020*	
C9	0.3966 (2)	0.0265 (5)	0.34022 (3)	0.0160 (3)	
H9	0.4878	−0.1048	0.3322	0.019*	
C10	0.2173 (2)	0.1348 (4)	0.32437 (3)	0.0142 (3)	
C11	0.1999 (2)	0.0697 (5)	0.29770 (3)	0.0160 (3)	
H11	0.3036	−0.0370	0.2904	0.019*	
C12	0.0306 (3)	0.1616 (5)	0.28197 (3)	0.0163 (3)	
H12	0.0205	0.1187	0.2643	0.020*	
C13	−0.1239 (2)	0.3191 (5)	0.29314 (3)	0.0161 (3)	
C14	−0.1123 (2)	0.3856 (5)	0.31941 (3)	0.0166 (3)	
H14	−0.2171	0.4903	0.3266	0.020*	
C15	0.0583 (2)	0.2938 (5)	0.33493 (3)	0.0167 (3)	
H15	0.0672	0.3383	0.3526	0.020*	

### Atomic displacement parameters (Å^2^)

**Table:**

	*U*^11^	*U*^22^	*U*^33^	*U*^12^	*U*^13^	*U*^23^
Cl1	0.0267 (2)	0.0276 (2)	0.01395 (17)	−0.00233 (17)	0.00251 (14)	−0.00137 (16)
Cl2	0.01366 (16)	0.02036 (19)	0.02387 (19)	−0.00204 (14)	0.00256 (13)	−0.00089 (15)
Cl3	0.01787 (17)	0.0229 (2)	0.0221 (2)	0.00123 (15)	−0.00347 (14)	0.00159 (16)
F1	0.0177 (5)	0.0353 (7)	0.0186 (5)	−0.0072 (5)	0.0003 (4)	−0.0023 (5)
O1	0.0187 (6)	0.0267 (7)	0.0183 (6)	0.0044 (5)	0.0036 (4)	−0.0029 (5)
C1	0.0135 (6)	0.0179 (7)	0.0163 (7)	0.0005 (5)	0.0023 (5)	0.0005 (6)
C2	0.0139 (6)	0.0184 (7)	0.0167 (7)	−0.0008 (5)	0.0008 (5)	0.0006 (6)
C3	0.0187 (7)	0.0173 (7)	0.0132 (6)	0.0012 (6)	0.0027 (5)	0.0010 (6)
C4	0.0160 (7)	0.0190 (7)	0.0163 (7)	0.0008 (6)	0.0046 (5)	0.0022 (6)
C5	0.0123 (6)	0.0139 (7)	0.0178 (7)	0.0008 (5)	0.0015 (5)	0.0007 (5)
C6	0.0139 (6)	0.0142 (7)	0.0149 (7)	0.0022 (5)	0.0013 (5)	0.0012 (5)
C7	0.0160 (6)	0.0151 (7)	0.0148 (7)	0.0002 (5)	0.0015 (5)	−0.0004 (5)
C8	0.0160 (7)	0.0159 (7)	0.0167 (7)	0.0022 (6)	0.0006 (5)	−0.0008 (6)
C9	0.0156 (6)	0.0156 (7)	0.0166 (7)	−0.0001 (5)	0.0014 (5)	0.0001 (6)
C10	0.0152 (6)	0.0126 (6)	0.0148 (6)	−0.0005 (5)	0.0017 (5)	−0.0001 (5)
C11	0.0177 (7)	0.0158 (7)	0.0148 (7)	0.0004 (6)	0.0036 (5)	−0.0008 (6)
C12	0.0201 (7)	0.0145 (7)	0.0141 (7)	0.0005 (6)	0.0010 (5)	0.0001 (5)
C13	0.0156 (6)	0.0154 (7)	0.0166 (7)	−0.0013 (6)	−0.0009 (5)	0.0016 (6)
C14	0.0157 (6)	0.0168 (7)	0.0175 (7)	0.0017 (6)	0.0028 (5)	−0.0003 (6)
C15	0.0169 (7)	0.0190 (7)	0.0143 (6)	0.0007 (6)	0.0023 (5)	−0.0006 (6)

### Geometric parameters (Å, °)

**Table:**

Cl1—C3	1.7190 (17)	C8—C9	1.341 (2)
Cl2—C5	1.7366 (17)	C8—H8	0.9300
Cl3—C13	1.7412 (17)	C9—C10	1.460 (2)
F1—C2	1.343 (2)	C9—H9	0.9300
O1—C7	1.224 (2)	C10—C11	1.402 (2)
C1—C2	1.380 (2)	C10—C15	1.403 (2)
C1—C6	1.395 (2)	C11—C12	1.388 (2)
C1—H1	0.9300	C11—H11	0.9300
C2—C3	1.390 (2)	C12—C13	1.391 (2)
C3—C4	1.386 (2)	C12—H12	0.9300
C4—C5	1.392 (2)	C13—C14	1.383 (2)
C4—H4	0.9300	C14—C15	1.389 (2)
C5—C6	1.394 (2)	C14—H14	0.9300
C6—C7	1.505 (2)	C15—H15	0.9300
C7—C8	1.469 (2)		
			
C2—C1—C6	120.37 (15)	C7—C8—H8	118.8
C2—C1—H1	119.8	C8—C9—C10	125.68 (16)
C6—C1—H1	119.8	C8—C9—H9	117.2
F1—C2—C1	119.49 (15)	C10—C9—H9	117.2
F1—C2—C3	119.28 (15)	C11—C10—C15	118.36 (15)
C1—C2—C3	121.23 (16)	C11—C10—C9	119.16 (15)
C4—C3—C2	119.29 (15)	C15—C10—C9	122.45 (15)
C4—C3—Cl1	121.14 (13)	C12—C11—C10	121.15 (16)
C2—C3—Cl1	119.55 (13)	C12—C11—H11	119.4
C3—C4—C5	119.29 (15)	C10—C11—H11	119.4
C3—C4—H4	120.4	C11—C12—C13	118.82 (15)
C5—C4—H4	120.4	C11—C12—H12	120.6
C4—C5—C6	121.82 (15)	C13—C12—H12	120.6
C4—C5—Cl2	116.97 (13)	C14—C13—C12	121.60 (15)
C6—C5—Cl2	121.17 (13)	C14—C13—Cl3	119.38 (13)
C5—C6—C1	117.98 (15)	C12—C13—Cl3	119.02 (13)
C5—C6—C7	124.71 (15)	C13—C14—C15	119.06 (16)
C1—C6—C7	117.29 (15)	C13—C14—H14	120.5
O1—C7—C8	124.03 (16)	C15—C14—H14	120.5
O1—C7—C6	118.75 (15)	C14—C15—C10	121.01 (16)
C8—C7—C6	117.19 (14)	C14—C15—H15	119.5
C9—C8—C7	122.36 (16)	C10—C15—H15	119.5
C9—C8—H8	118.8		
			
C6—C1—C2—F1	−179.66 (16)	C5—C6—C7—C8	49.0 (2)
C6—C1—C2—C3	0.1 (3)	C1—C6—C7—C8	−132.33 (17)
F1—C2—C3—C4	−179.28 (16)	O1—C7—C8—C9	7.5 (3)
C1—C2—C3—C4	0.9 (3)	C6—C7—C8—C9	−174.50 (16)
F1—C2—C3—Cl1	1.8 (2)	C7—C8—C9—C10	177.37 (16)
C1—C2—C3—Cl1	−177.94 (14)	C8—C9—C10—C11	173.51 (18)
C2—C3—C4—C5	−0.9 (3)	C8—C9—C10—C15	−8.3 (3)
Cl1—C3—C4—C5	177.97 (14)	C15—C10—C11—C12	0.4 (3)
C3—C4—C5—C6	−0.2 (3)	C9—C10—C11—C12	178.70 (16)
C3—C4—C5—Cl2	177.69 (14)	C10—C11—C12—C13	−0.4 (3)
C4—C5—C6—C1	1.2 (3)	C11—C12—C13—C14	0.0 (3)
Cl2—C5—C6—C1	−176.57 (13)	C11—C12—C13—Cl3	179.54 (14)
C4—C5—C6—C7	179.86 (16)	C12—C13—C14—C15	0.2 (3)
Cl2—C5—C6—C7	2.1 (2)	Cl3—C13—C14—C15	−179.27 (14)
C2—C1—C6—C5	−1.2 (3)	C13—C14—C15—C10	−0.2 (3)
C2—C1—C6—C7	−179.91 (16)	C11—C10—C15—C14	−0.2 (3)
C5—C6—C7—O1	−132.87 (19)	C9—C10—C15—C14	−178.37 (17)
C1—C6—C7—O1	45.8 (2)		

### Hydrogen-bond geometry (Å, °)

**Table:**

*D*—H···*A*	*D*—H	H···*A*	*D*···*A*	*D*—H···*A*
C8—H8···Cl2	0.93	2.81	3.1164 (16)	101
C9—H9···O1	0.93	2.57	2.878 (2)	100
